# Bioinformatic screening of human ESTs for differentially expressed genes in normal and tumor tissues

**DOI:** 10.1186/1471-2164-7-94

**Published:** 2006-04-26

**Authors:** Abdel Aouacheria, Vincent Navratil, Audrey Barthelaix, Dominique Mouchiroud, Christian Gautier

**Affiliations:** 1 Laboratoire de Biométrie et Biologie Evolutive, CNRS UMR 5558, Université Claude Bernard Lyon 1, 69622 Villeurbanne Cedex, France; 2 Aptanomics, 181-203, avenue Jean Jaurès 69007 Lyon, France; 3 Current address: Apoptosis and Oncogenesis Laboratory, IBCP, UMR 5086 CNRS-UCBL, IFR 128, Lyon, France

## Abstract

**Background:**

Owing to the explosion of information generated by human genomics, analysis of publicly available databases can help identify potential candidate genes relevant to the cancerous phenotype. The aim of this study was to scan for such genes by whole-genome *in silico *subtraction using Expressed Sequence Tag (EST) data.

**Methods:**

Genes differentially expressed in normal versus tumor tissues were identified using a computer-based differential display strategy. Bcl-xL, an anti-apoptotic member of the Bcl-2 family, was selected for confirmation by western blot analysis.

**Results:**

Our genome-wide expression analysis identified a set of genes whose differential expression may be attributed to the genetic alterations associated with tumor formation and malignant growth. We propose complete lists of genes that may serve as targets for projects seeking novel candidates for cancer diagnosis and therapy. Our validation result showed increased protein levels of Bcl-xL in two different liver cancer specimens compared to normal liver. Notably, our EST-based data mining procedure indicated that most of the changes in gene expression observed in cancer cells corresponded to gene inactivation patterns. Chromosomes and chromosomal regions most frequently associated with aberrant expression changes in cancer libraries were also determined.

**Conclusion:**

Through the description of several candidates (including genes encoding extracellular matrix and ribosomal components, cytoskeletal proteins, apoptotic regulators, and novel tissue-specific biomarkers), our study illustrates the utility of *in silico *transcriptomics to identify tumor cell signatures, tumor-related genes and chromosomal regions frequently associated with aberrant expression in cancer.

## Background

Large-scale transcriptome analysis of genes that are differently expressed in tumor tissues compared to their normal counterparts is an important route to the identification of candidates that could play a role in human malignancies. A number of techniques, ranging from differential display and nucleic acid subtraction to serial analysis of gene expression, expression microarrays and gene chips, have been used to the discovery of such aberrantly expressed cancer-related genes [1]. The well-established differential screening technology, that allows for the simultaneous comparison of multiple gene expression levels between two samples differing in tissue type and pathological state, has been the more extensively applied. This simple and powerful method could be performed either experimentally or, since late 1999, digitally using expression databases. The *computer-based *differential display methodology, also referred to as '*in silico *subtraction' or 'electronic northern' [2-7], could identify transcripts preferentially expressed or repressed in the tumor context by comparing cancerous libraries (present in publicly available databases) against the remaining libraries. Strikingly, only few attempts were made to apply *in silico *transcriptomics to genome-wide and multi-tissue screening of cancer genes [8-10]. Thus, given the continuous expansion of the EST databases, both in terms of sequence and source diversity, updated and independent transcriptomic analyses are permanently needed.

In this study, we mined EST libraries for genes differentially expressed in normal and tumor tissues by using a novel computational approach, with the assumption that both the up- and down-regulated pools might contain genes involved in tumorigenesis. This strategy identified differential expression profiles and cancer candidate genes which may be useful in future cancer research. Higher expression of the anti-apoptotic protein Bcl-xL in liver cancer specimens compared to normal liver was confirmed by immunoblot analysis. Strikingly, we found that most cancer-associated changes in gene expression corresponded to genes that were actually downregulated or repressed. The chromosomes and chromosomal regions most frequently associated with aberrant expression changes in tumor versus normal cells were also determined. This analysis suggests that, although genes differentially expressed in cancerous libraries are distributed throughout the genome, chromosomal 'hot spots' of candidate genes could be identified.

## Results

### Identification of differentially expressed genes between normal and cancer tissues

Genes differentially expressed in tumor libraries compared to their normal counterparts are likely to play important roles in cancer etiology or could constitute relevant genetic markers for cancer diagnosis. Here, we have performed *in silico *differential display to identify novel and known cancer-associated genes by comparing all the libraries representing tumors to the corresponding normal libraries for each tissue type. Details about the data mining procedures are presented in Table 1. In order to be able to compare expression levels between normal and tumor state, we compared EST counts from non-normalized, non-subtracted cDNA libraries. To overcorrect for the false positive rate, we decided to perform the highly conservative Bonferroni correction. Using this procedure, a total of 673 genes showed differential expression in tumor versus normal libraries by a factor of 10 or higher (Additional File 1: 'Upregulated candidates complete list', and Additional File 2: 'Downregulated candidates complete list'), with about one third being up-regulated (299) and the remaining being down-regulated (539). The *in silico *subtraction also resulted in the identification of 181 and 336 genes predicted to be present or absent in the tumor types compared to normal tissues, respectively. Because these EST clusters were identified either in normal or tumor libraries, it was not possible to derive their expression ratio, so we decided to present them as separated tables (Additional File 3: 'Tumor specific candidates complete list', and Additional File 4: 'Normal specific candidates complete list'). However, these two groups of genes have been fused to the 'up-regulated' and 'down-regulated' pools in the subsequent analyses. All in all, a sum of 112 novel transcripts was also found (i.e. sequences for which no description was available at the time of the study). Noteworthy, *in silico *subtraction identified 14.5 % (154/1060) previously studied genes involved in oncogenesis, based on a list of ~ 2500 genes compiled as previously described [11]. Since the fraction of such reference genes in our initial data set was 7.5 % (2401/31800), our data mining protocol expectedly lead to a significant enrichment in cancer genes (p value = 2.2 10^-16^; exact Fisher test). These previously characterized and well-studied genes include the *p57*^*KIP*2 ^and *p19*^*INK*4*d *^cyclin inhibitors, and the *ras-GAP*, *c-fos*, *ret *and *myc *oncogenes. Last, in order to independently verify the validity of the EST-based tissue profiles, SAGE data were used to give an indication of the tissue distribution of our transcripts in normal tissues. While SAGE results specified by tissue type converged with the analysis of ESTs for 65,4 % (197/301), 53,9 % (91/169), and 53,2 % (93/171) of the examined hits in the 'down', 'up' and 'normal-specific' groups respectively, i.e. precisely in the classes where an expression in the normal condition was expected, this percentage decreased to 37,7 % (46/122) for the 'tumor-specific' group of transcripts.

### Cancer candidate gene analysis

**Figure 1:**
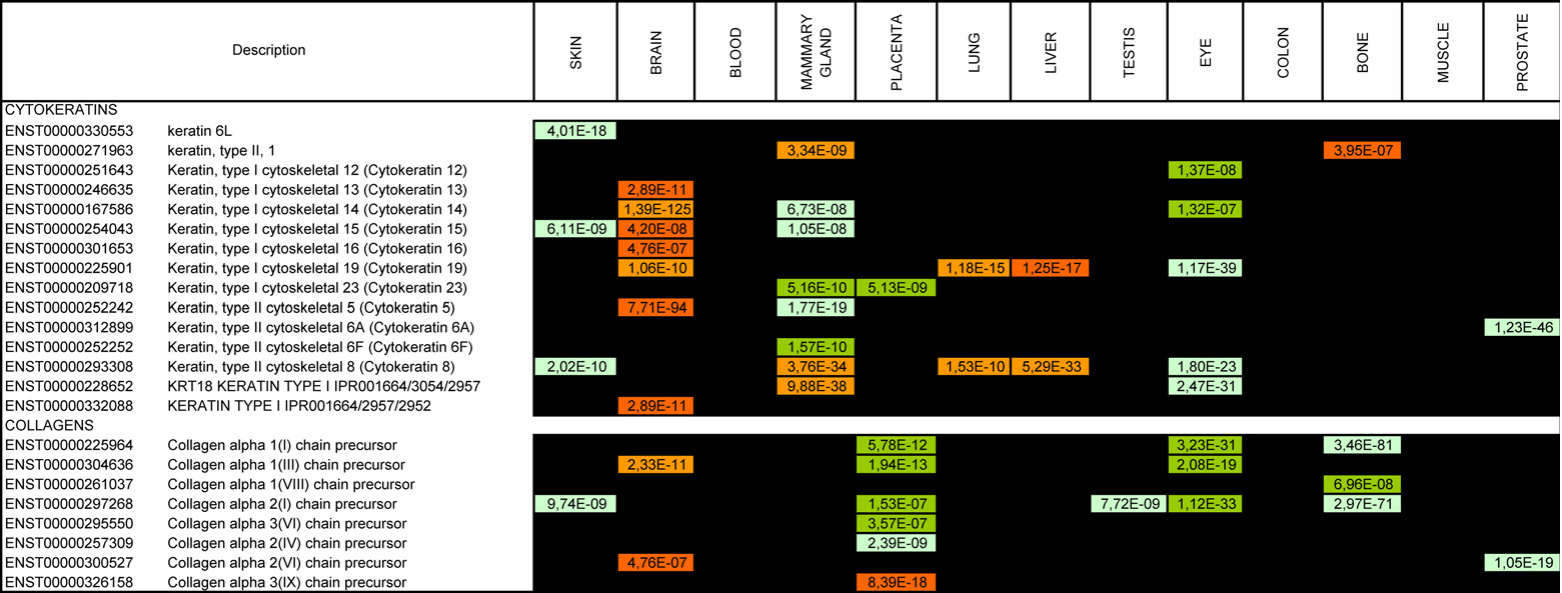
**Patterns of differential expression for collagen and cytokeratin genes in multiple normal and tumor tissues**. The data are shown in a table format, in which rows represent individual genes and columns represent individual normal tissue. The color in each cell reflects the differential expression level of the corresponding gene in a particular tissue. A four color code was used to represent gene induction and repression in cancer libraries (dark green: 'normal-specific', i.e. not expressed in tumor libraries; light green: downregulated in tumor libraries; orange: upregulated in tumor libraries; red: 'tumor-specific'). If there was no significant change in gene expression between normal and tumor libraries or in case of missing/excluded data, the gene was given in a black color. The number inside the colored cells indicates the statistical significance (*p*-value < 0.01 after Bonferroni correction). See additional information for the full data.

**Figure 2:**
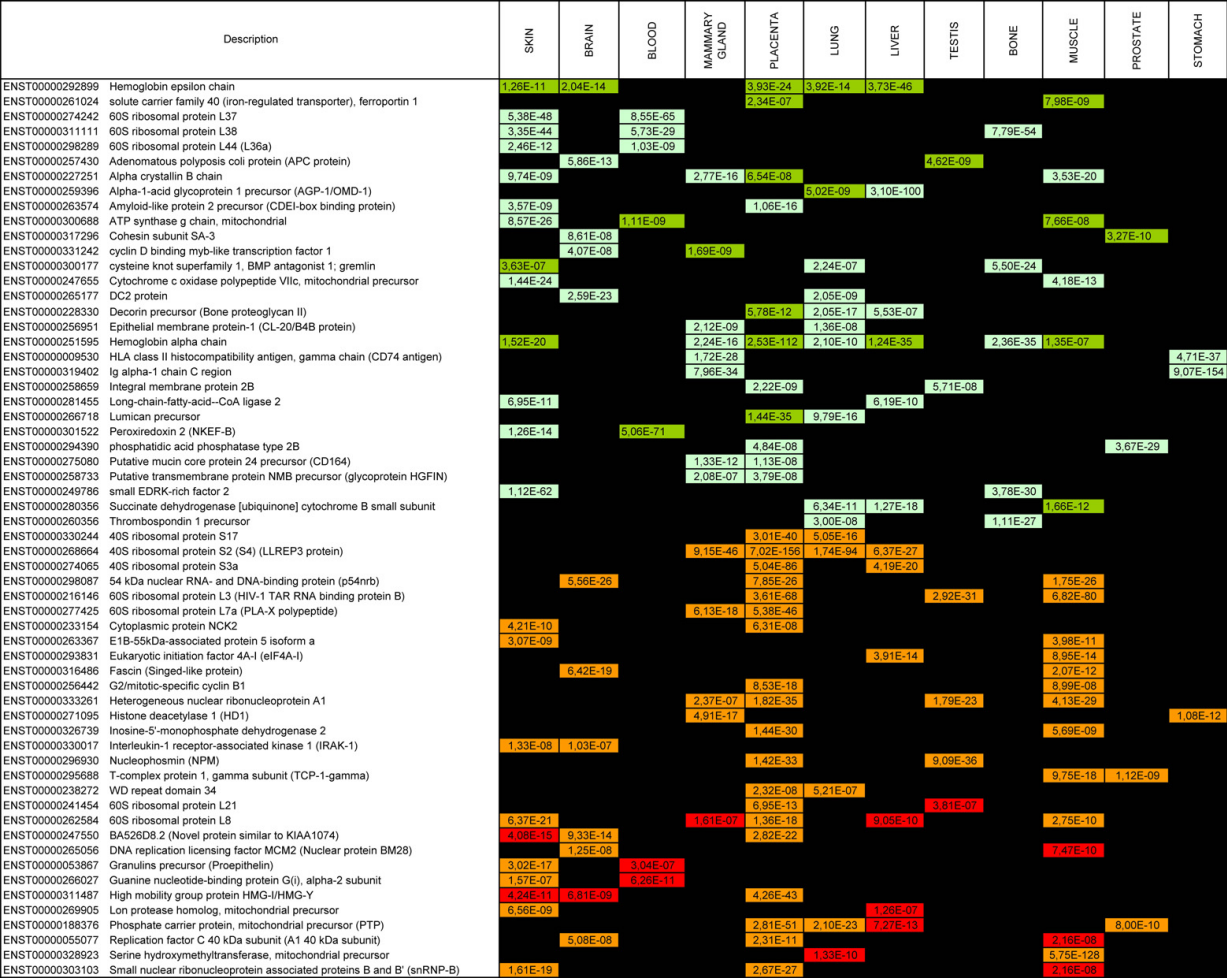
**Genes whose transcripts varied significantly and consistently in abundance in at least two different tissues**. Thirty genes were selected in each class of differential expression (upregulated, downregulated, tumor-specific or absent in tumors). The results are shown for twelve tissues. The legend is the same as in Figure 1. See additional information for the full data.

**Figure 3:**
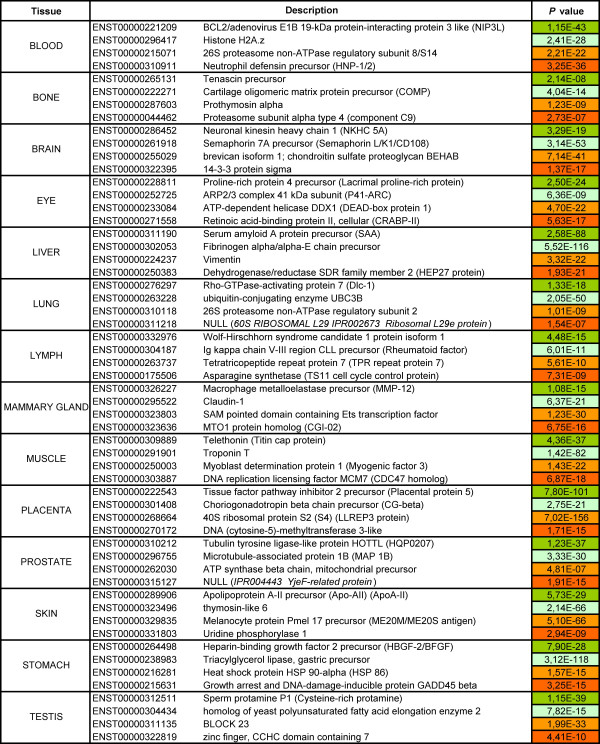
**Genes whose transcripts exhibited tissue-specific differential expression in normal versus tumor libraries**. This figure is a compilation of genes that appear to be differentially expressed in only one of the 15 studied tissues. The results are shown for fourteen tissues. The color code is the same as in Figure 1. See additional information for the full data.

The first general observation that could be made from our results is that a same gene could be either up-regulated or repressed according to the tumor cell type, allowing identification of tissue-specific gene expression profiles in tumor versus normal cells. For instance, among the set of candidates with differential expression in cancer, we observed a massive down-regulation of several collagen alpha chain genes (but not beta chain genes) in various tumor tissues, including decreased expression of collagen alpha 2(I) (also termed *col1A2*) in skin, placenta, testis, eye and bone (see Figure 1). Interestingly, *col1A2 *has been reported as a tumor suppressor gene that could inhibit *ras*-induced oncogenic transformation [12,13]. Apart from collagens, other types of proteins that could be used as useful biomarkers include cytokeratins (CK). CK are particularly interesting epithelia specific intermediate filaments because their degradation gives rise to soluble fragments, measurable in the blood of patients and capable of cancer monitoring [14]. Our results show that a total of 13 CK genes were differentially expressed between normal and malignant cells in 9 different tissues (Figure 1), allowing tissue-specific expression profiling (e.g. specific expression of CK 5, 13 and 16 in tumor brain). Additionally, in line with previous microarray data [15], we found that hair-specific type II keratin was overexpressed in breast tumors compared to normal breast. We further determined that over the 190 genes which displayed aberrant expression in more than one tissue, 131 were "deregulated" in the same way (either up- *or *down, Figure 2 and Additional File 5: 'Consistent candidates in multiple tissues'). Included in this list of 'consistent' candidates are 13 transcripts encoding different ribosomal components, in accordance with the increasing body of evidence from the literature that correlates changes in the protein synthesis machinery with cancer [16-18]. Specific signatures for ribosomal genes could be determined, e.g. downregulation of the genes encoding 60S ribosomal L37, L38 and L44 in libraries prepared from tumor skin and tumor blood, whereas placental cancer libraries appear to be specifically enriched in transcripts encoding 40S ribosomal S2, S3 and S17. As depicted in Figure 3 (for the full data set, see Additional File 6: 'Tissue specific candidates'), some genes display a tissue-specific pattern of differential expression in tumor types, thus making them candidates for specific diagnostic markers. Among these 114 genes differentially expressed in only one tissue are *14-3-3 sigma *in brain tumors and *Bnip3L *in blood. This latter gene, belonging to the Bcl-2 family of apoptotic regulators, has been described as a potential tumor suppressor [19,20]. Last, it is worth noting that a novel member of the methyltransferase enzyme family (ENST00000270172), that contains clear transcriptional repressors [21,22], was found to be specifically overexpressed in placental tumors.

Taken together, these results suggest that EST data could be successfully mined to provide digital profiles of differential gene expression at the full genome level between normal and cancerous tissues. Our lists of transcriptional signatures might help to select candidate markers in cancer genetics or potential targets for therapy.

### Increased expression of Bcl-xL in liver tumors

**Figure 4:**
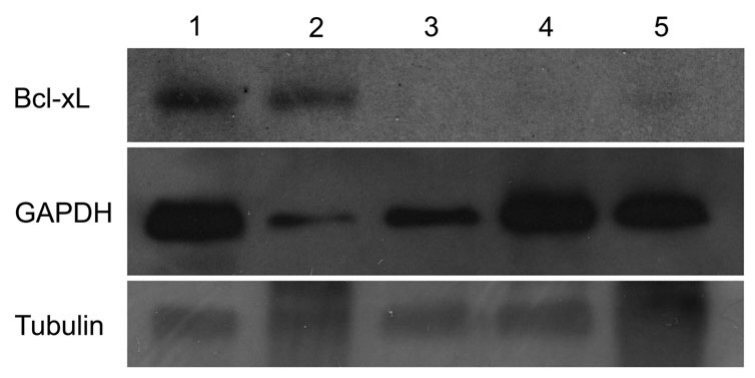
**Western blot analysis of Bcl-xL expression in human normal and tumoral liver**. Lane 1: hepatocellular carcinoma (male, age 65); lane 2: adenocarcinoma (male, age 52); lane 3: cholangiocellular carcinoma (male, age 46); lane 4: normal liver (male, age 24); lane 5: normal placenta (female, age 24). Bcl-xL immunoreactivity (26 kD) was observed in two out of three liver cancer samples (lanes 1–3). Normal samples (lane 4–5) had no signal for Bcl-xL expression. Note that GADPH protein levels varied between normal tissues and cancer liver specimens and did not correlate with the mRNA levels predicted by the computer-based screen. The expression of tubulin was used as control for equal protein loading.

Bcl-2 family member Bcl-xL (Bcl2-associated X membrane protein) was selected for confirmation by immunoblotting due to its plausible biological role in cancer susceptibility. Moreover, both EST and SAGE results indicated that Bcl-xL was poorly expressed in normal liver, while abundant in other tissues (both normal and cancerous, data not shown), suggesting that this apoptotic regulator could constitute a good marker for liver cancer progression. As depicted in Figure 4, western blot analysis confirmed overexpression of Bcl-xL in a subset of human liver cancer specimens (hepatocellular carcinoma, adenocarcinoma but not cholangiocellular carcinoma) compared to normal liver (and placenta).

### Identification of chromosome locations of differential gene expression in cancer

We next sought to analyze the chromosomal distribution of the genes which were over-expressed or repressed in tumor tissues. To this end, we mapped the previously identified genes showing significant differential expression between normal and tumor tissues along human chromosomes according to their banding, in order to build cancer-oriented transcriptome maps. To avoid possible biases due to chromosome length (e.g. chromosome Y as an obvious case) or different chromosomal gene densities, we computed the percentage of candidate genes against the total number of genes present on a particular chromosome or banding (see Table 2).

First, our results show that some chromosomes appear to be more active than others (Table 2A), with, for instance, chromosomes 15, 19 and Y being rarely involved in cancer-related gene expression changes compared to chromosomes 4 and 6. As expected from the results of Table 1, most chromosomal regions associated with changes in expression levels actually correspond to gene inactivation patterns in cancer cells (373 up-regulated versus 744 down-regulated hits), striking examples of cancer-associated inactivation of gene expression being chromosome 17 and chromosome 3. While most tissues (14/16) were clearly subject to these cancer-associated gene inactivation patterns (especially lung, eye, colon, prostate and stomach), two tissues (tumor blood and liver) did not follow this trend. Chromosomal regions displaying at least five hits were further listed and this rough analysis was sufficient to detect 11 and 29 regions of clustering of up- and down-regulated genes, respectively (Table 2B). We found previously identified chromosomal regions associated with either tumor amplicon (e.g. 12q13) or deleted (e.g. 11p15) regions in tumors [23-26]. Interestingly, some of the chromosomal locations which were identified show tissue specificity, e.g. 12q13.3 in muscle. Moreover, in some cases, candidate genes could be contiguous or clustered in limited banding intervals. For instance, 19q13 is associated in tumor tissues of placental origin with complete extinction of eight clustered genes, namely pregnancy-specific beta-1-glycoproteins PSG-1, -2, -3, -4, -5, -6, -9 and -11. These genes belonging to the carcinoembryonic antigen family encode the major placental proteins found in maternal circulation during pregnancy [27,28].

In conclusion, in addition to providing differential expression profiles for individual genes, our EST-based procedure identified discrete regions on specific chromosomes that are enriched in genes deregulated in cancer libraries.

## Discussion

Owing to advances in biotechnology and bioinformatics, researchers can now capture "molecular portraits" of various particular cancers using gene chips or SAGE data. These methods provide information on tens of thousands of genes simultaneously, and some variations in genes might be directly related to the cancer phenotype [1,29]. As multi-dimensional analysis of EST data is analogous to microarray experiments, we used the virtual differential display methodology to identify genes differentially expressed in normal versus tumor tissues. Our comprehensive approach gives an overview of numerous candidate genes which may be useful as improved biomarkers for diagnosis or as targets for developing novel treatment methods. For instance, EST-based formulation of collagen, integrin or cytokeratin expression profiles may have potential as a diagnostic aid for the detection of both tumor formation and development. Noteworthy, for discovery of tumor-associated molecules, it may be beneficial to use a combination of various digital differential display procedures and experimental data on gene expression. This is illustrated by the identification of prostate-specific Ets factor as a novel marker for breast cancer both computationally [8,30] (and this study) and experimentally [30-32].

General limitations of EST-based strategies, which have been abundantly discussed elsewhere [4,33,34], include poor sequencing depth of the libraries, uncertainty concerning the origin of the samples, and differences in library sizes. In addition, analysis of tumor-related differential expression patterns of individual transcripts may have specific drawbacks. For example, cancer cells often proliferate more rapidly than adjacent normal cells and it is possible that, in some cases, the observed changes in transcript abundance may reflect a response to increased proliferation rather than transformation *per se*. One related problem is that many cell types are often pooled together during the preparation of EST libraries. Given that most cancers start as growths of single cells, the lack of cell-type specific libraries is a major limiting factor of the method. Lastly, the determined variations in transcript expression may not correlate with similar variations in the abundance of the encoded protein, highlighting the need to experimentally test the computer-based predictions either by western blotting or immunohistochemistry. Our validation result showing that Bcl-xL protein expression was markedly increased in hepatocellular carcinoma and liver adenocarcinoma suggests that this Bcl-2 family member represent a potential marker for progression of a subset of liver cancers. Analysis of Bcl-xL immunoreactivity in more liver cancer specimens is needed to enhance the reliability of this finding. However, as it correlates with previous results [35-37], and in view of the pro-survival effect of Bcl-xL, we hypothesize that Bcl-xL overexpression could confer specific protection from death to several types of liver cancer cells compared to their healthy counterparts. If true, modulation of Bcl-xL expression level and/or activity might represent an interesting strategy to optimize the efficacy of chemotherapeutic agents in this particular tissue, as liver cancer represents a significant source of morbidity and mortality worldwide [38].

Aside from the proposal of potential diagnosis markers and targets for future cancer research, a more theoretical perspective of our study is the identification of critical factors that could influence differential gene expression levels in normal versus cancer cells, including genomic landscape features, e.g. levels of polymorphisms, chromosome breakpoints, gene density, GC content and chromatin methylation status. In this regard, although we cannot rule out the possibility of unidentified biases in our data mining procedure, our result showing a higher frequency of gene inactivation patterns in tumor tissues is intriguing, and sheds light on the importance of understanding the molecular mechanisms of negative gene regulation in cancer.

## Conclusion

The final outcomes of the present work are identification of chromosomal regions frequently associated with aberrant expression in cancer libraries, description of differential expression profiles, and listing of cancer candidate genes (e.g. Bcl-xL) which may be useful as tissue-specific biomarkers for cancer diagnosis or as targets for anticancer research.

## Methods

### Data preparation

We have used an EST-based pipeline to scan for differential gene expression levels between normal and tumor states. Human ESTs from dbEST [39] (October 2004 release) were first extracted using the ACNUC sequence retrieval system [40]. ESTs were classified according to their UNIGENE library features [41] (October 2004). For each EST in dbEST, we extracted the accession code of the EST and the tissue or organ from which the EST library has been made. The tissue type was retrieved from the line containing 'Tissue_type', 'Tissue description', 'Organ' or 'Keyword'. This parsing approach stored no data when the tissue information did not appear in these fields, or in case of typographical errors or ambiguous aliases. ESTs that were labeled as coming from an unspecified tissue (e.g. 'mixed', 'pooled organs', 'cell line') or from a mixture of specified tissues, were discarded. The eVOC ontology [42] (October 2004) for anatomical sites and pathology types was then used to classify the libraries through a number of criteria such as tissue origin and pathological context including tumor state. This well-accepted hierarchical vocabulary provided us with a mean to determine when a specific tissue was part of an organ and when a specific label was part of the 'tumoral' state. A total of 5135 'tumor' and 2503 'normal' (i.e. non-pathological) libraries were catalogued. Our approach to EST clustering used the human genome as a reliable guide. ENSEMBL RNAs [43] annotated on human genome assembly (release 16.3) were used as a backbone for the clustering of dbEST sequences using MEGABLAST (alignment length = 100 bp and similarity = 95%) [44]. In order to avoid paralogous false positive assignation, only best EST hit matches were subsequently selected. RNA clustering of ESTs in both normal and tumor tissues was the starting point for digital differential analysis of gene expression.

### Computer-based differential display procedure

The cDNA libraries were categorized into non-normalized or normalized/subtracted libraries by screening for the appropriate keywords in the original annotation of the respective dbEST entries (in the 'Keyword' and 'Library treatment' fields). All libraries for which none of the keywords were found were defined as being non-normalized. After removal of normalized and subtracted EST libraries, we created pools of equivalent EST libraries, i.e. libraries derived from the same tissue type and state (normal and tumor). Differential screening analysis was accomplished for a considered tissue when both normal and tumor pools displayed at least 10,000 ESTs. A total of 15 distinct paired tissue pools (blood, bone, brain, colon, eye, liver, lung, lymph, mammary gland, muscle, placenta, prostate, skin, stomach, testis) representing approximately 1.5 million ESTs were therefore retained for the whole genome screening. Differential screening was performed for each tissue type individually using EST counts from tumors and corresponding normal counterparts. The relative expression of one particular gene in a tissue was characterized by the ratio of the number of ESTs matching this gene to the total number of ESTs sequenced in the respective tissue. As such 'gene expression profiles' were derived from 'normal' and 'tumor' libraries, it was possible to build 2 × 2 contingency tables and then to apply the Fisher exact test against the null hypothesis that there was no association between a particular gene and the tumoral state. A *p*-value was determined for statistical significance and, because multiple tests were performed, a Bonferroni correction was applied on each pairs in order to reduce the false positive rate and to perform candidate gene sorting. Statistically significant hits showing at least 10-fold differences were compiled. Four classes of genes were defined, namely (i) genes displaying significantly higher expression levels in tumor tissues ('up-regulated' genes); (ii) genes displaying significantly lower expression levels in tumor tissues ('down-regulated' genes); (iii) genes expressed in tumor but not in normal tissues ('tumor-specific' genes); (iv) genes absent in the tumor types compared to normal tissues. Apart from the genes displaying absolute differences between normal and tumor condition, a ratio based on EST abundance in both conditions was computed to estimate the expression fold change for up- and down-regulated genes. Cytogenetic map position of the hits was inferred using ENSEMBL data (release 16.3). The pattern of expression of the differentially expressed transcripts (n = 1190, as determined by the EST analysis) in normal tissues was independently assessed by comparison to SAGE results obtained on the SAGE Genie website [45] and processed as previously described [46]. A total of 141 (non-tumoral) libraries containing more than 20,000 tags were partitioned into 19 normal tissues. The expression pattern of 13,435 transcripts was determined. Eight tissues (blood, brain, colon, liver, lung, mammary gland, placenta and prostate) were unambiguously mapped to the tissue terms used in the EST data mining procedure. From this sample, we queried as to which candidate transcripts associated with differential expression in a particular tissue (on the basis of the EST predictions) was expressed in the corresponding normal tissue (according to the SAGE data). Information on differential expression was also gained from reference to primary literature. As this effort corresponded to a manual task particularly unfitted to the large number of candidate genes presented here, we limited the analysis to the "up-regulated" and "down-regulated" lists related to the liver and breast tissues. We found that 54.2% (for liver) and 41.7% (for breast) of the annotated candidates identified through our computer-based screen were consistent with previously published data (see Additional files 1 and 3). The differential display procedure and other analytical steps were developed with R [47]. Expression and genomic data were stored in a local PostgreSQL database (GeMCore) [48] using PERL and Java script.

### Western blot analysis

Nitrocellulose membrane was from Euromedex (Souffelweyersheim, France). The membrane was immunoblotted with anti-human Bcl-xL antibody (1:1 000 dilution, BD Pharmingen), and then with anti-mouse IgG antibody conjugated to horseradish peroxidase (1:5 000 dilution, Dako). Protein bands were revealed using enhanced chemiluminescence kit (ECL, Amersham). The membrane was stripped according to manufacturer's instructions and reprobed with anti-glyceraldehyde-3-phosphate dehydrogenase (GAPDH) monoclonal antibody (1:1 000 dilution) and with anti-alpha-tubulin (1:1000 dilution, Sigma) to correct for differences in protein loading.

## Authors' contributions

AA designed the study, performed the immunoblot assays, analyzed the data and drafted the manuscript. VN developed the algorithm for the differential display procedure, processed the SAGE data, participated in the data analyses and reviewed the manuscript. AB provided the antibodies and participated in the western blotting experiments. DM and CG provided funding and supervision for the work. All authors read and approved the final manuscript.

## Supplementary Material

Additional File 1 Upregulated candidates complete list. Upregulated genes in tumor tissues (complete list). Hits displaying at least a 10-fold increase in tumor-derived libraries compared to their normal tissue counterpart are shown. Chromosomal locations for each hit were inferred from Ensembl cytogenetic map. Hits were sorted by p value (exact Fisher's test; *p *< 0.05, Bonferroni corrected), ranked by expression ratio and ordered by tissue. Both known and novel ('NULL') transcripts are listed. 'Y': 'Yes'; 'ND': non-determined. Pubmed ID (PMID) is given for annotated candidate transcripts whose differential expression was documented in previously published data. '*': *in silico *studies. Click here for file

Additional File 2 Downregulated candidates complete list. Downregulated genes in tumor tissues (complete list). Hits displaying at least a 10-fold decrease in tumor-derived libraries compared to their normal tissue counterpart are shown. Chromosomal locations for each hit were inferred from Ensembl cytogenetic map. Hits were sorted by p value (exact Fisher's test; *p *< 0.05, Bonferroni corrected), ranked by expression ratio and ordered by tissue. Both known and novel ('NULL') transcripts are listed. 'Y': 'Yes'; 'ND': non-determined. Pubmed ID (PMID) is given for annotated candidate transcripts whose differential expression was documented in previously published data. '*': *in silico *studies. Click here for file

Additional File 3 Tumor specific candidates complete list. Complete list of genes absent from normal tissues and present in tumor types. Tumor-specific hits are shown. Chromosomal locations for each hit were inferred from Ensembl cytogenetic map. Hits were sorted by p value (exact Fisher's test; *p *< 0.05, Bonferroni corrected) and ranked by tissue origin. Both known and novel ('NULL') transcripts are listed. 'Y': 'Yes'; 'ND': non-determined. Click here for file

Additional File 4 Normal specific candidates complete list. Summary of genes absent from tumor types and present in normal tissues. Genes absent in the tumor types compared to normal tissues is shown. Chromosomal locations for each hit were inferred from Ensembl cytogenetic map. Hits were sorted by p value (exact Fisher's test; *p *< 0.05, Bonferroni corrected) and ranked by tissue origin. Both known and novel ('NULL') transcripts are listed. 'Y': 'Yes'; 'ND': non-determined. Click here for file

Additional File 5 Consistent candidates in multiple tissues. Genes whose transcripts varied significantly and consistently in abundance in at least two different tissues. Click here for file

Additional File 6 Tissue specific candidates. Genes whose transcripts exhibited tissue-specific differential expression in normal versus tumor libraries. Click here for file

## Figures and Tables

**Table 1:** Overview of the EST-based data mining strategy. Screening for differentially expressed genes between normal and cancer tissues. EST counts in each analytical step. Total number of EST clusters in each class (upregulated, downregulated, tumor-specific or absent in tumors) was determined after Bonferroni corrected exact Fisher test.

Total RNA (Ensembl)	~ 31,800
Total ESTs (after clustering)	~ 3.3 10^6^
Total clusters	26,601
Total up-regulated EST clusters	227
Total down-regulated EST clusters	473
Total EST clusters specific to tumors	173
Total EST clusters absent in tumors	308

**Table 2:** Chromosomal regions of differential gene expression in cancer. (A) Number of hits, i.e. number of genes with differential expression per chromosome, is depicted. 'Up' and 'down' mean chromosomal regions with increased and decreased tumor expression, respectively. '%' represents the percentage of candidate genes against the total number of genes present on the chromosome. (B) Chromosomal regions found to be associated with at least 5 hits in the digital subtraction analysis are shown ('banding'). '%' represents the percentage of hits for a particular chromosomal banding against the total number of genes present in the same banding. Chromosomal bandings marked in bold correspond to previously identified regions associated with either tumor amplicon ('Up' column) or deleted ('Down' column) regions in tumors.

	**A**						**B**			
Chromosome	Up		Down		Up+Down		Up		Down	
	
	Total Hits	%	Total Hits	%	Total Hits	%	Banding	%	Banding	%

1	33	1.02	54	1.68	87	2.70			**1q21**.3 **1q32**.1	3.12 3.20
2	11	0.75	28	1.90	39	2.65			2p11.2 2p13.3 2q35	6.66 9.26 4.55
3	11	0.55	58	2.89	69	3.43	**3p21**.31	4.03	**3p21**.31	3.14
4	38	2.26	46	2.73	84	4.99			4q13.3	9.37
5	4	0.67	8	1.35	12	2.02			**5q33**.1	6.58
6	14	1.22	36	3.15	50	4.37	**6p21**.31	6.33		
7	11	1.05	19	1.81	30	2.86			**7q21.**3 **7q22**.1	8.93 3.32
8	18	1.25	30	2.08	48	3.34				
9	31	1.69	39	2.13	70	3.82			**9q34**.11 **9q34**.3	3.60 3.60
10	5	1.05	10	2.10	15	3.15				
11	30	1.32	46	2.03	76	3.36			**11p15**.1 **11p15**.4 **11p15**.5 11q12.2	7.81 2.56 5.88 7.81
12	25	1.14	61	2.78	86	3.91	**12q13**.13 **12q13**.3	4.80 10.12	12q13.13	7.20
13	19	1.76	21	1.94	40	3.70				
14	5	1.27	8	2.03	13	3.29	14q11.2	2.59	**14q11**.2 **14q32**.33	2.59 8.89
15	7	0.84	16	1.93	23	2.77				
16	19	1.08	40	2.28	59	3.36	**16p11**.2	3.10	**16p11**.2 **16q12**.2	3.10 9.80
17	9	0.74	38	3.14	47	3.88	**17q21**.2 **17q25**.3	5.71 2.81	**17q21**.2 17q23.3	5.00 8.06
18	12	0.86	31	2.22	43	3.08				
19	14	0.80	29	1.65	43	2.44	**19p13**.3	2.43	**19p13**.11 **19q13.**2 **19q13**.31 **19q13**.33	2.15 3.69 7.04 3.14
20	10	0.56	38	2.13	48	2.69			20q13.12	2.92
21	8	0.70	25	2.18	33	2.87	**21q22**.3	3.12	**21q22**.3	3.75
22	17	1.20	31	2.19	48	3.40				
X	21	1.59	31	2.34	52	3.93	**Xq28**	4.76		
Y	1	0.64	1	0.64	2	1.27				
